# The Placental Protein Syncytin-1 Impairs Antiviral Responses and Exaggerates Inflammatory Responses to Influenza

**DOI:** 10.1371/journal.pone.0118629

**Published:** 2015-04-01

**Authors:** Jorge M. Tolosa, Kristy S. Parsons, Philip M. Hansbro, Roger Smith, Peter A. B. Wark

**Affiliations:** 1 Mothers and Babies Research Centre and Hunter Medical Research Institute, The University of Newcastle, Newcastle, NSW, Australia; 2 Centre for Asthma and Respiratory Disease and Hunter Medical Research Institute, The University of Newcastle, Newcastle, NSW, Australia; 3 Department of Respiratory and Sleep Medicine, John Hunter Hospital, New Lambton, NSW, Australia; University of Geneva, SWITZERLAND

## Abstract

**Background:**

Pregnancy increases susceptibility to influenza. The placenta releases an immunosuppressive endogenous retroviral protein syncytin-1. We hypothesised that exposure of peripheral monocytes (PBMCs) to syncytin-1 would impair responses to H1N1pdm09 influenza.

**Methods and Findings:**

Recombinant syncytin-1 was produced. PBMCs from non-pregnant women (n=10) were exposed to H1N1pdm09 in the presence and absence of syncytin-1 and compared to responses of PBMCs from pregnant women (n=12). PBMCs were characterised using flow cytometry, release of interferon (IFN)-α, IFN-λ, IFN-γ, IL-10, IL-2, IL-6 and IL-1β were measured by cytometric bead array or ELISA. Exposure of PBMCs to H1N1pdm09 resulted in the release of IFN-α, (14,787 pg/mL, 95% CI 7311-22,264 pg/mL) IFN-λ (1486 pg/mL, 95% CI 756-2216 pg/mL) and IFN-γ (852 pg/mL, 95% CI 193-1511 pg/mL) after 48 hours. This was significantly impaired in pregnant women (IFN-α; p<0.0001 and IFN-λ; p<0.001). Furthermore, in the presence of syncytin-1, PBMCs demonstrated marked reductions in IFN-α and IFN-λ, while enhanced release of IL-10 as well as IL-6 and IL-1β.

**Conclusions:**

Our data indicates that a placental derived protein, syncytin-1 may be responsible for the heightened vulnerability of pregnant women to influenza.

## Introduction

The advantage of genetic diversity has ensured the success of sexual reproduction in evolution, but in placental mammals it comes with the inherent dilemma that the maternal immune system must tolerate the existence of the semi-allogenic foetus/placenta that expresses both self (maternal) and non-self (paternal) antigens[1]. It is also clear that to prevent maternal rejection of the foetus and to sustain a successful pregnancy, potent mechanisms of immune regulation must be activated, such as suppression of T cell activation and function[2,3] and the generation of suppressor T regulatory (TReg) cells that function in an antigen specific manner[4]. Recent advances have demonstrated the importance of co-ordinating immune responses between pathogen detection (non-self antigens) by the innate immune system and activation or suppression of adaptive immune responses. In this regard dendritic cells (DCs) are an essential link in coordinating these immune responses in health and in pregnancy[5,6]. The placenta also appears to play an important though poorly understood role in promoting the immunotolerant state[2].

The human placenta is unique amongst tissues in having the highest expression of human endogenous retroviruses. These are evolutionary fossils, which at some ancient time point have infected germ line cells. Their ongoing high level expression in the placenta implies that they have imparted an evolutionary advantage for the host[7]. Syncytin-1 is a naturally occurring placental protein that is encoded by one of these retroviruses and has been shown to fuse human cytotrophoblast cells and promote syncytialisation that is crucial for placental formation[8]. Recently we also determined that human syncytin-1 released from the placenta suppresses maternal immune responses demonstrating it’s potential to play a role in maternal/foetal tolerance[9].

Maternal immune tolerance may come at the cost of increased susceptibility to infection, with pregnant women at greater risk of infection from intracellular bacteria and viruses[10]. The emergence of a new pandemic strain of swine influenza in 2009 (herein referred to as H1N1pdm09) recently refocussed attention on this issue. In this pandemic 50% of the women who died were pregnant. This is a startling statistic that emphasises that pregnant women are at increased risk of developing infection with influenza and are also more likely to develop severe disease[11]. This increase in mortality and morbidity is also observed during seasonal influenza; with numerous studies reporting increased rates of influenza-induced respiratory hospitalisations in pregnant compared to non-pregnant women[12–15]. While several comorbidities have been identified that predisposed to susceptibility to H1N1pdm09 infection, pregnancy is the most intriguing as it imparts a temporary susceptibility of the maternal immune system to infection. To further understand this phenomenon, it is of particular note that those pregnant women with severe H1N1pdm09 infection presented with an acute syndrome of severe respiratory compromise and systemic inflammation, associated with aberrant immune responses. This is characterised by intense acute inflammation (increased levels of the cytokine IL-6), but also reduced antiviral T-helper (TH)-1 responses (reduced IFN-γ) and heightened suppressor T cell signals (IL-10)[16]. The implications of these observations are that the development of a maternal state of immune tolerance may particularly impair their immune response to novel pathogens such as H1N1pdm09.

To further explain the susceptibility of pregnant women to influenza we hypothesised that the human endogenous retroviral envelope protein syncytin-1 suppresses maternal cell mediated immune responses to influenza viruses, impairing antiviral DCs, promoting the development of suppressor TReg cells and inhibiting the development of robust anti-viral TH-1 immune responses.

## Methods

### Subjects

To assess the naïve innate response to virus we recruited women who had never been vaccinated to H1N1pdm09. Twelve pregnant women (non-vaccinated) (PNV) and 10 healthy non-pregnant women (non-vaccinated) (NPNV), recruited from October 2012-October 2013, participated in this study. Pregnant women were recruited from the John Hunter Hospital (JHH) antenatal clinics and non-pregnant women were recruited by advertisement. Inclusion criteria for all participants were females of child-bearing age (18–40 years). Pregnant women were recruited from any trimester. Women were excluded if they had any concomitant chronic medical illness, drug or alcohol dependence, currently smoking, if they had asthma or other respiratory conditions or if they had cold/flu symptoms within the four weeks prior to sample collection. Written informed consent was obtained from all participants prior to sample collection and ethics approval was obtained from the Hunter New England Human Research Ethics Committee and the University of Newcastle Research Ethics Committee. Pregnant and non-pregnant women attended a single study visit at which baseline characterisation was assessed and venepuncture was performed.

### Virus Stocks

A strain of 2009 pandemic swine flu (H1N1 A/Auckland/3/2009) was obtained from the World Health Organization (WHO Melbourne) in 2010. Viral stocks were propagated in Madin-Darby Canine Kidney (MDCK) cells (ATCC, Manassas, VA, USA), as previously described[17–19]. Stock viral concentrations were measured using plaque assays; which determines live virions based on plaque forming units per ml (pfu/ml)[17–19]. Ultra-violet (UV) inactivation of live viruses was achieved by placing live viruses directly under a UV lamp (254nm) for 3hrs. Successful inactivation was confirmed by plaque assays.

### Generation of Syncytin-1 Ectodomain

Syncytin-1 recombinant ectodomain protein was produced and purified following previously stated methods[9,20]. Syncytin-1 recombinant ectodomain was cloned into the pET-28b vector (Novagen) using BspH1, Nco1 and Xho1 restriction enzymes. For scale up production, the protein was expressed in *E*. *coli* BL21 (DE3) cells (Stratagene) in 10 L of Luria Broth culture media (Sigma) and expression was induced with 1mM IPTG (Sigma). The protein was extracted from the soluble cytoplasmic material using BugBuster Master Mix (Merck Millipore, Novagen). The clarified supernatant was purified twice on nickel-coupled Hitrap IMAC HP columns (three Hitrap IMAC HP 5 mL columns (GE) connected in series). After concentration and buffer exchange using Amicon Ultra centrifugal filter 10K (Millipore), the protein was pre-purified on a preparative Sephadex G75 (Sigma-Aldrich) 2.5 x 100 cm column (Econo-Column, Bio-Rad) with a flow rate of 0.7 mL/min. The bioactive oligomeric fraction was then further purified on an analytical Sephadex G75 1.5 x 120 cm column (Econo-Column, Bio-Rad) with a flow rate of 0.2 mL/min. in 25 mM HEPES, 150 mM NaCl, pH7.4. The protein was concentrated using an Amicon Ultra centrifugal filter 10K (Millipore) and filter sterilized (0.2 μm). To ensure no endotoxin was present in the preparation, the syncytin-1 stock was tested using the Limulus Amebocyte Lysate (LAL) QCL-1000 kit (LONZA) following the manufacturer’s instructions.

### Syncytin-1 Stock Preparation

The protein stock was stored at-20°C at a concentration of 100 μM in 25 mM HEPES, 150 mM NaCl, pH7.4. A 20 μM working solution was prepared by diluting 1 volume of 100 μM stock with 4 volumes of buffer (25 mM HEPES, 150 mM NaCl, pH7.4). Fifty μL of 20 μM syncytin working solution was added to form a final concentration of 1 μM.

### Peripheral Blood Mononuclear Cell (PBMC) Isolation and Culture

PBMCs were isolated from whole blood by density centrifugation using Ficoll-Paque PLUS (GE Healthcare Uppsala, Sweden) and resuspended in Roswell Park Memorial Institute media (RPMI; Invitrogen, Australia Pty Limited) in 10% foetal bovine serum (FBS;SAFC Biosciences, Lenexa, Kansas, USA). PBMCs were cultured in 24 well plates at a final concentration of 2.0x10^6^ cells/ml (NUNC, Denmark). Cells were stimulated with 1 μM syncytin-1 for 2hrs before virus stimulation. After this the cells were stimulated with H1N1 at MOI 0.1 (2.0x10^5^pfu/ml), or cultured in media alone for 48hrs, 35°C and 5% CO2. These concentrations were based on the ability of the virus to induce maximal IFN production with minimal cell death at 48hrs (S3A-C Fig.). After culture, cellular suspensions were centrifuged at 550xg for 10 min. Cell lysates were stored in Buffer RLT (QIAGEN Pty Ltd Doncaster, VIC) and supernatants were stored at-80°C for subsequent analysis.

### ELISA

IFN-λ1 protein was measured from culture supernatants by ELISA following the manufacturer’s instructions (R&D Systems, MN, USA) with analysis using on a Fluorostar Optima microplate reader (BMG Labtech, Ortenberg, Germany).

### Cytometric Bead Array (CBA)

IFNγ, IFNα, IL-10, IL-6, and IL-1β were measured in culture supernatants using CBA (BD Bioscience CA, USA). The samples were run on a BD FACS Canto II Flow Cytometer and analysed with BD FCAP Array Software (BD Bioscience CA, USA), according to the manufacturer’s instructions.

### Antibodies for Flow Cytometry

There were two flow cytometry panels used; a DC and a lymphocyte panel. These panels were further subdivided into cytokine measures to identify which cells were releasing cytokines as well as markers of cell activation. The fluorochrome-conjugated antibodies used in the DC cytokine panel were: Fixable Viability Stain 450, CD45 BV510, HLA-DR BV605, CD56 PerCP-Cy5.5, CD123 PE, CD14 Pe-Cy5, CD19 PE-Cy5, CD11c AF700, CD3 APC-H7, IL-6 FITC, IFN-γ PE-Cy7, IFN-α AF647 and IL-10 PE-CF594. Antibodies used in the DC activation marker panel were: Fixable Viability Stain 450, CD45-BV510, CD56 PerCP-Cy5.5, CD123 PE, CD14 Pe-Cy5, CD19 PE-Cy5, CD11c AF700, CD3 APC-H7, HLA-DR APC, CD80 PE-Cy7, CD86 PE-CF594 and HLA-A, B, C FITC. Antibodies used in the lymphocyte cytokine panel were: Fixable Viability Stain 450, CD45 BV510, CD56 BV605, CD4 PerCP-Cy5.5, FoxP3 PE, CD25 APC, CD8 AF700, CD3 APC-H7, IL-6 FITC, IFN-γ PE-Cy7 and IL-10 PE-CF594. Antibodies used in the lymphocyte activation marker panel were: Fixable Viability Stain 450, CD45 BV510, CD56 BV605, CD4 PerCP-Cy5.5, FoxP3 PE, CD25 APC, CD8 AF700, CD3 APC-H7, CD103 PE-Cy7, CTLA-4 PE-CF594 and GITR AF488. All were from BD Bioscience, CA, USA except CD14, CD19, GITR and CD103 (ebioscience, USA). Appropriate controls were used in all experiments.

### Flow Cytometry General Gating for all PBMC Experiments

To further elucidate the cell types that were producing the cytokines of interest, additional intracellular cytokine staining was performed on cells from two (n = 2) non pregnant, not vaccinated women. All samples were run on a BD FACS ARIA Flow Cytometer and FlowJo software (Tree Star Inc, USA). Gating strategies for excluding doublets, cell clumps, dead cells, intracellular and surface marker staining were based on instructions from BD Bioscience. Doublets and cell clumps were excluded at the same time as gating the PBMC population by creating scatter plots for FSC-H vs FSC-A, FSC-A vs FSC-W, SSC-A vs FSC-A, and CD45+/FVS450 negative. The cell populations that fell within these four plots were considered the single cell viable PBMC population. A total of 5x10^5–^1x10^6^ events were then collected within these intersected gates.

Isolated PBMCs were first cultured for 44hrs before adding a 1:10 dilution of the protein blocker, brefeldin A (BD Bioscience, CA, USA) and then cultured for a further 4 hours. After a total of 48 hours, PBMCs were centrifuged at 300xg for 10 minutes and resuspended in PBS buffer at a final concentration of 1x10^6^/ml PBMCs. Fixable Viability Stain 450 protocol was performed according to the manufacturer’s instructions. PBMCs were then stained for either the DC panel or the lymphocyte panel for cytokines or activation markers.

### Expression of Cytokines in DCs and Lymphocytes

The DC panel employed followed the BD Bioscience cytofix/cytoperm manufacturer’s instructions. PBMCs were stained with CD123 for pDCs and CD11c for mDC. PBMCs were also stained with CD14 and CD19 to exclude monocytes and B cells, respectively. To determine the DC populations within the live single cell (FVS450-) PBMC populations (CD45+), B cells and monocytes were first excluded. This was done by constructing a scatter plot for CD14/CD19 vs CD3. Lymphocytes were then gated as the CD3- population. To exclude basophils and NK cells, an HLA-DR vs CD11c plot was created and DCs were considered HLA-DR positive. From the HLA-DR positive cells, mDC cells were considered the CD11c+ population whilst pDCs were considered the CD123+ from a CD123 vs CD11c scatter plot. IL-10, IL-6, IFN-γ and IFN-α intracellular cytokine expression of each DC population was determined by plotting each and measuring the percentage of positive cells of the parent population.

The lymphocyte panel employed followed the BD Bioscience Transcription factor buffer set manufacturer’s instructions. PBMCs were stained with CD4 for T helper cells, CD8 for cytotoxic T cells, CD56 for NK cells and FoxP3/CD25 for T regulatory cells. To determine the T cell populations within the live single cell (FVS450-) PBMC populations (CD45+) a scatter plot for CD56 vs CD3 was created and T lymphocytes were then gated as the CD3+ population. T helper cells were considered the CD4+ population whilst cytotoxic T cells were considered the CD8+ from a CD8 vs CD4 scatter plot. The CD4+ cells were then placed on a FoxP3 vs CD25 scatter plot and T regulatory cells were considered FoxP3/CD25+. NK cells were gated as CD56+. IL-10, IL-6 and IFN-γ cytokine expression of each lymphocyte population were determined by plotting each and measuring the percentage of positive cells of the parent population.

### Expression of Activation Markers in DCs and Lymphocytes

For the DC panel, to measure activation marker expression on pDCs and mDCs, PBMCs were stained with antibodies for CD86, HLA-DR, CD80 and HLA-A, B, C. Following incubation for 30 minutes at 4°C, PBMCs were washed twice with flow stain buffer, centrifuged and resuspended at a final volume of 350μl in flow stain buffer. The same gating strategy was followed as above. However, in this case, to determine the percentage of pDCs/mDCs positive for CD86, HLA-DR, CD80 and HLA-A, B, C, scatter plots were constructed for each marker against the CD123+ (pDC) or CD11c+ (mDC) population, gated on the parent population. Mean fluorescence intensity was also measured for each activation marker.

For the lymphocyte panel, to measure activation marker expression on T lymphocytes, PBMCs were stained with antibodies for GITR, CTLA-4 and CD103. The lymphocyte panel used followed the BD Bioscience Transcription factor buffer set manufacturer’s instructions. The same gating strategy was followed as above. However, in this case, to determine the percentage of CD4/CD8/Treg/NK cells positive for GITR, CTLA-4, and CD103, scatter plots were constructed for each marker against the CD4+ (CD4), CD8+ (CD8), CD4+/FoxP3+/CD25+ (Treg), CD3-/CD56+ (NK cells) population, gated on the parent population. Mean fluorescence intensity was also measured for each activation marker.

### Statistical Analyses

Data are expressed as mean ± standard error of mean (SEM) for twelve subjects (n = 12) with two replicates for each subject for pregnant women and ten subjects (n = 10) with two replicates for each subject for non-pregnant women (except for the flow cytometry analysis which was carried out on n = 2 non pregnant women). Statistical significance was assessed by one-way analysis of variance (ANOVA), post-hoc analysis was performed using the Holm-Sidak’s correction using GraphPad Prism Software. A P value of <. 05 was considered significant. For NPNV, H1N1 was compared to H1N1+Syncytin, media alone and to H1N1 for PNV. Also for NPNV, syncytin alone was compared to media. A comparison was made between NPNV H1N1+Syncytin and PNV H1N1. Also for PNV, H1N1 was compared to H1N1+Syncytin and media alone.

## Results

### PBMCs from non-pregnant women have a robust IFN response to H1N1pdm09 infection, which is reduced in pregnant women

**Fig 1:**
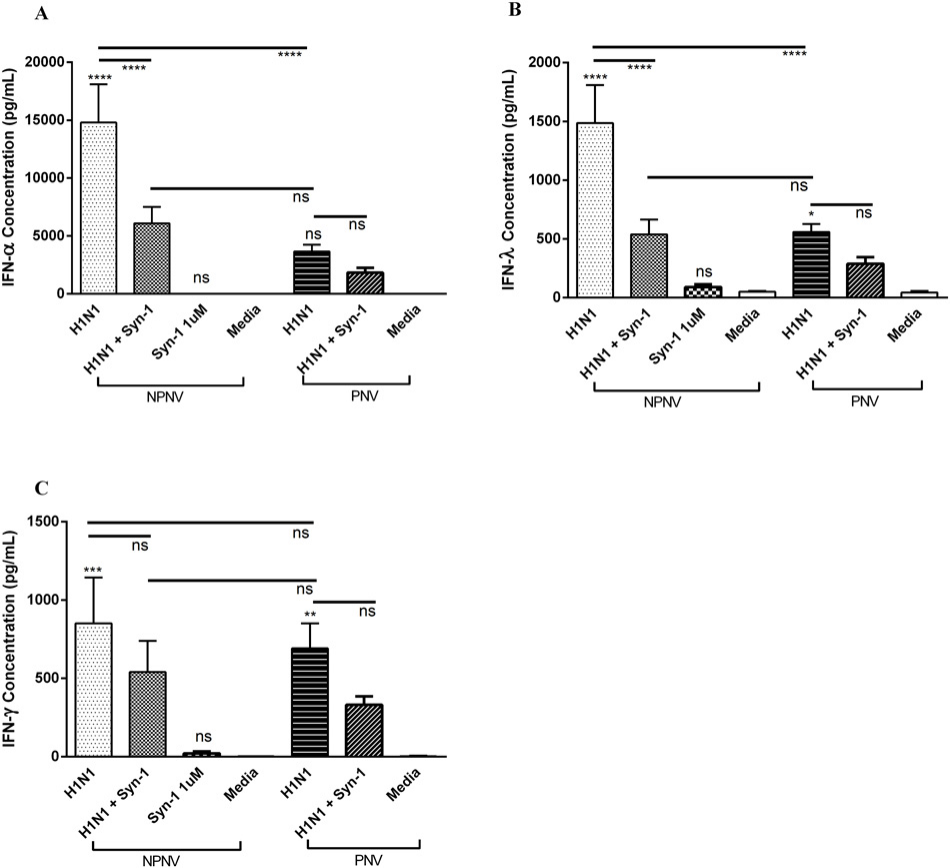
PBMCs from non-pregnant women have a robust IFN response to H1N1pdm09. The response of PBMCs from pregnant and non-pregnant women to H1N1pdm09 was assessed after 48 hours of exposure. PBMCs from non-pregnant women were pre-exposed to syncytin-1 (1 μM). PBMCs were infected with H1N1 MOI 0.1 for 48hrs. Levels of IFN-α (A), IFN-λ (B) and IFN-γ (C) in cell culture supernatants were measured by cytometric bead array (IFN-α and IFN-γ) or ELISA (IFN-λ). NPNV = Non-pregnant and non-vaccinated n = 10, PNV = Pregnant and non-vaccinated n = 12. ANOVA (Holm-Sidak) **** *P* < 0.0001, *** *P* < 0.001, **P < 0.01, * *P* < 0.05

PBMCs from pregnant and non-pregnant women were cultured with a strain of influenza isolated during the 2009 swine flu pandemic (H1N1pdm09) or cultured in media only. PBMCs from non-pregnant women demonstrated a robust IFN-α (14,787 pg/mL, 95% CI 7311–22,264 pg/mL) and IFN-λ (1486 pg/mL, 95% CI 756–2216 pg/mL) response to H1N1pdm09 (Fig. 1A-C,) compared to media alone after 48hours of exposure. Conversely, PBMCs from pregnant women, following H1N1pdm09 stimulation, produced significantly less IFN-α (Fig. 1A) (3661pg/mL, 95% CI 2382–4939 pg/mL) and IFN-λ (Fig. 1B) (558 pg/mL, 95% CI 407–710 pg/mL) compared to PBMCs from non-pregnant women (p<0.0001 and p<0.001, respectively). However, this was not the case with IFN-γ (691 pg/mL, 95% CI 340–1043 pg/mL) (Fig. 1C).

### Treatment of PBMCs from non-pregnant women treated with syncytin-1 prior to infection with H1N1pdm09 reduced IFN responses, to levels similar to those in PBMCs from pregnant women

PBMCs from non-pregnant women were pre-treated with 1 μM syncytin-1 and then cultured with H1N1pdm09 or media only. This dose of syncytin-1 was selected by performing a dose response on a range of biologically relevant doses (1nM to 5 μM). 1μM had the ability to reduce IFN-α and IFN-λ and induce IL-10 (S1 A-C Fig.). Cytotoxicity of syncytin-1 has also previously been measured and confirmed to have no effect on cell viability or cytokine release[9]. Furthermore, the syncytin-1 preparation technique did not result in any endotoxin in the final protein. Using size exclusion chromatography we were able to separate syncytin-1 into two major peaks, one corresponding to a low level oligomerization and another as a multimeric complex. The multimeric peak corresponds to the bioactive conformation of the protein and has been used in all experiments. The low level oligomerization peak was also used but found not to be bioactive. The addition of syncytin-1 significantly reduced IFN-α (6083 pg/mL, 95% CI 2848–9319 pg/mL) and IFN-λ responses (539 pg/mL, 95% CI 255–823 pg/mL) (p<0.001 Fig. 1A-B). In fact treatment with syncytin-1 induced a response from these cells that mirrored what was seen in PBMCs from pregnant women with infection alone (H1N1 PNV) (Fig. 1A-C).

### PBMCs from non-pregnant women treated with syncytin-1 prior to infection have a heightened IL-10 response

**Fig 2:**
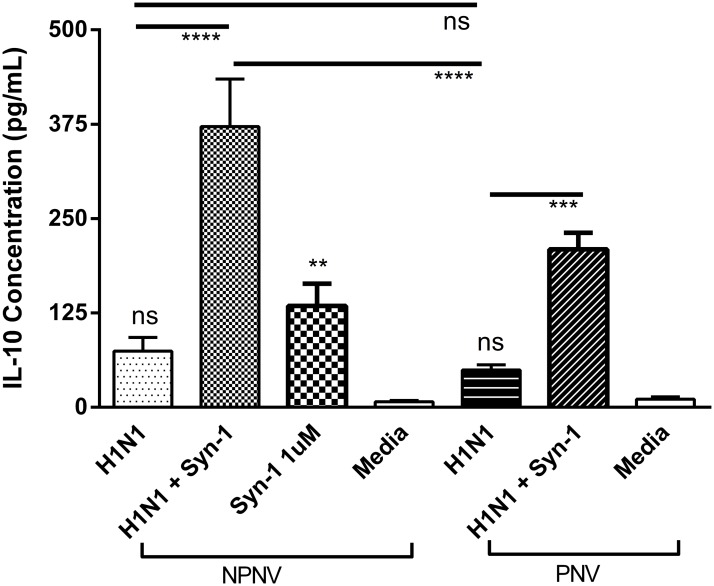
PBMCs from non-pregnant women treated with syncytin-1 prior to infection have a heightened IL-10 response. PBMCs were infected with H1N1 MOI 0.1 for 48hrs with and without syncytin-1 (1 μM). Levels of IL-10 were measured in culture supernatants by cytometric bead array. Non-pregnant PBMC demonstrated a heightened IL-10 response when exposed to H1N1pdm09 infection and syncytin-1. NPNV = Non-pregnant and non-vaccinated n = 10, PNV = Pregnant and non-vaccinated n = 12. ANOVA (Holm-Sidak) **** *P* < 0.0001, *** *P* < 0.001, **P < 0.01.

H1N1pdm09 infection alone did not induce an IL-10 response from either pregnant or non-pregnant PBMCs when compared to media (Fig. 2) (75 pg/mL, 95 CI 34–114 pg/mL). However, there was a substantial increase in IL-10 in non-pregnant PBMCs with pre-treatment with syncytin-1, p<0.0001 (372 pg/mL, 95% CI 230–514 pg/mL).

### The plasmacytoid DCs are the source of the released IFN-α/λ and IL-10 in response to H1N1pdm09

**Fig 3:**
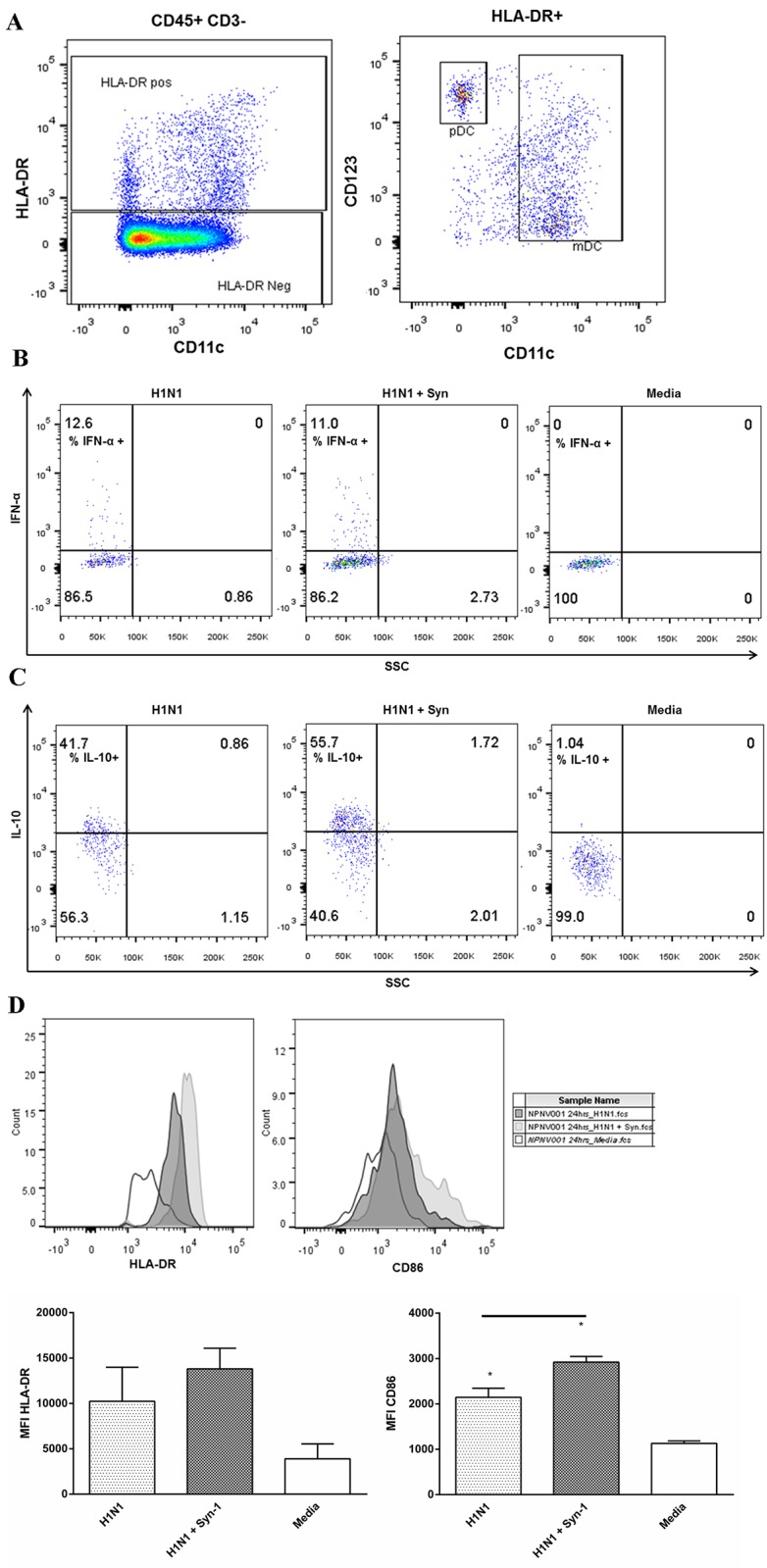
Flow cytometric analysis of PBMCs showing that pDCs are responsible for the release of IFN-α and IL-10. PBMCs were infected with H1N1pdm09 MOI 0.1 for 48hrs with and without syncytin-1. The cells were then subjected to surface and intracellular cytokine staining and flow cytometry to determine cell populations and cytokine release. The pDC population was determined using CD3-/CD45+ cells which were also HLA-DR+ and CD123+ (A). This population was analysed for cytokine release using intracellular staining and the percentage of cells positive for IFN-α (B) and IL-10 (C) was calculated. Surface staining of pDCs for the activation markers HLA-DR and CD86 (D) was also measured. NPNV = Non-pregnant and non-vaccinated n = 2, ANOVA (Holm-Sidak) * *P* < 0.05.

We then used flow cytometry and surface staining to identify the cells responsible for the observed responses. The DC lineage was identified as CD3-/HLA-DR+, and PBMCs were further characterised as plasmacytoid DCs (pDCs) (CD123+) or myeloid DCs (mDCs) (Fig. 3A).

Intracellular cytokine staining of the pDC population showed that pDCs were responsible for the release of IFN-α. The pDCs had increases in the percentage of IFN-α positive cells in the presence of H1N1pdm09 compared to media (Fig. 3B Panel 1 and 3). While the addition of syncytin-1 and H1N1pdm09, reduced pDC IFN-α expression (Fig. 3B Panel 2). Furthermore, intracellular cytokine staining of the CD4 lymphocytes showed that the CD4+ population was responsible for the release of IFN-γ, with the percentage of positive cells reduced in the presence of syncytin-1 (S2A Fig.), though, at the early time-point of assessment, the release was small.

Intracellular cytokine staining of the pDC population also showed that these cells were responsible for the increased release of IL-10 in the presence of H1N1pdm09 when compared to media (Fig. 3C Panel 1 and 3). While the combination of syncytin-1 and H1N1pdm09, further increased pDC expression of IL-10 (Fig. 3C Panel 2). Although the majority of the IL-10 was from pDCs, there was also a small amount expressed by CD4 TReg cells (S2B Fig.).

To confirm that these pDCs were active, surface expression of CD86 and HLA-DR were measured. Infection with H1N1pdm09 increased the expression of HLA-DR and CD86 (Fig. 3D). This expression was further amplified with syncytin-1 pre-treatment.

### PBMCs from non-pregnant women treated with syncytin-1 prior to infection have a heightened release of IL-6 and IL-1β

**Fig 4:**
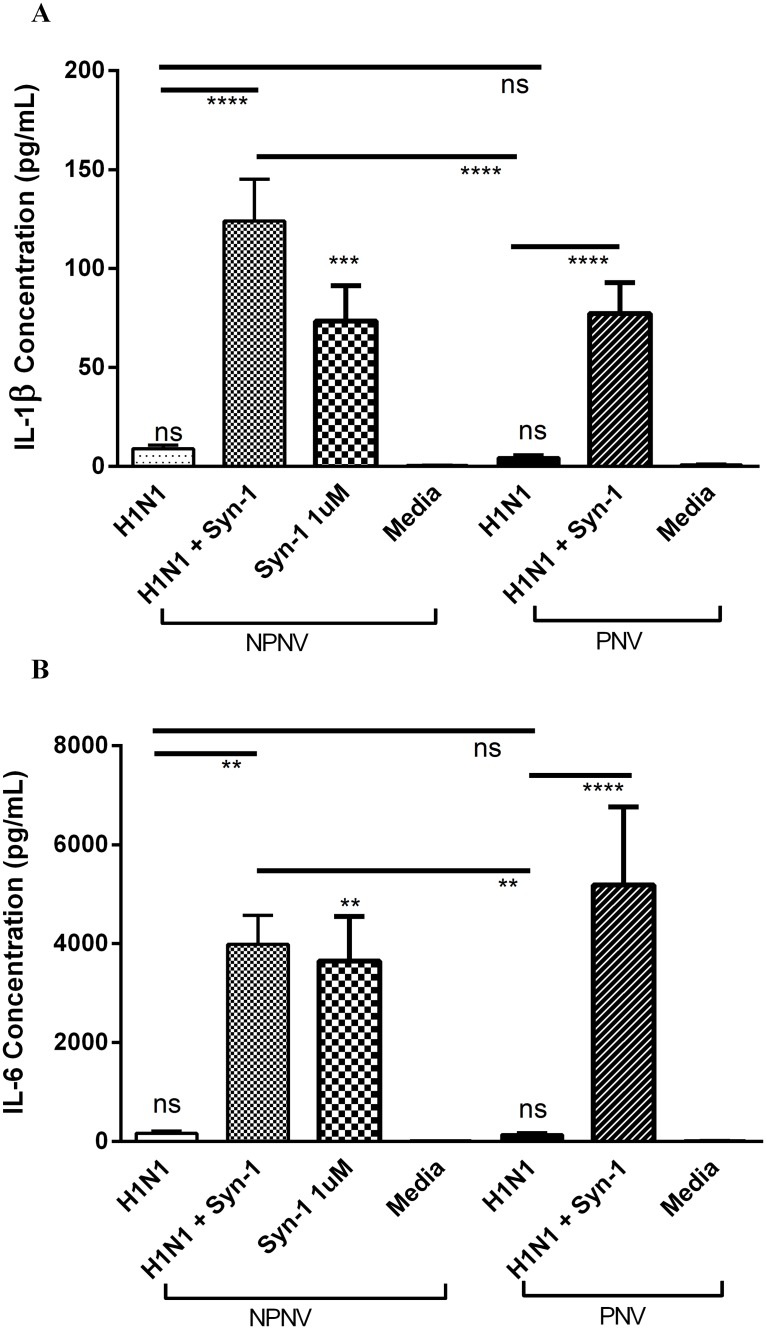
Pregnant and non-pregnant PBMC inflammatory response to H1N1pdm09 infection and the effect of Syncytin-1. PBMCs were infected with H1N1pdm09 MOI 0.1 for 48hrs with and without syncytin-1. Levels of IL-1β (A), and IL-6 (B) in culture supernatants were measured by ELISA and cytometric bead array. NPNV = Non-pregnant and non-vaccinated n = 10, PNV = Pregnant and non-vaccinated n = 12. ANOVA (Holm-Sidak) **** *P* < 0.0001, *** *P* < 0.001, **P < 0.01.

H1N1pdm09 infection alone did not induce release of IL-1β or IL-6 PBMCs from either pregnant or non-pregnant women when compared to media. However, there was a substantial increase in IL-1β from non-pregnant PBMCs pretreated with syncytin-1 (p<0.0001 Fig. 4A) (124 pg/mL, 95% CI 76–172 pg/mL). Further, there was also an exaggerated IL-6 release from non-pregnant PBMCs pretreated with syncytin-1 (p<0.0001 Fig. 4B) (3985 pg/mL, 95% CI 2659–5310 pg/mL). Both of these responses were significantly elevated compared to those seen in pregnant PBMCs with infection alone (p<0.0001) (4.3 pg/mL, 95% CI 1–7 pg/mL;140 pg/mL, 95% CI 63–217 pg/mL respectively).

## Discussion

Impaired immune responses to intracellular pathogens, such as influenza represent an ongoing and important risk for otherwise healthy pregnant women. For the first time we have demonstrated that a placental derived protein, syncytin-1, impairs early innate immune responses to influenza. Exposure of PBMCs from NPNV women to H1N1pdm09, resulted in a robust antiviral response, characterised by the release of IFN-α and IFN-λ. However this response is considerably reduced in PBMCs from PNV women. Furthermore exposure of PBMCs from NPNV women to syncytin-1 remarkably alters their normal antiviral response; impairing the release of IFN-α/λ, and promoting the release of IL-10 as well as the pro-inflammatory cytokines IL-1β and IL-6. We went on to demonstrate that this early antiviral response is predominantly mediated by pDCs, confirming our previous data that there are impaired early innate immune responses to influenza in pregnancy[21], but now demonstrating that this can be induced by exposure of pDCs to syncytin-1.

Sequences of retroviral origin make up a substantial part of the mammalian genome. Analysis of the human genome revealed that around 8% of human DNA is derived from retrovirus-like elements[22]. Human Endogenous Retroviruses (HERVs) are derived from exogenous retroviruses, which, at some ancient time-point, have infected germline cells[7,23] and, therefore, are passed on to all human cells. A HERV envelope gene belonging to the HERV-W family and encoding a protein expressed in the syncytiotrophoblast was first described in 1999[24] and was denoted syncytin-1[8]. Syncytin-1 was shown to fuse human cytotrophoblast cells and may be a key factor in regulating syncytialization during placental formation[8,25]. In addition to the fusogenic property of syncytin-1 and its role in syncytialization, the presence of an immunosuppressive region (ISD) in the amino acid sequence was also identified[8,24]. Syncytin-1 is normally produced and secreted by the placenta in exosomes. During pregnancy, maternal plasma exosomal protein concentrations average 0.61±0.14; 0.94±0.41 and 1.4±0.11 mg/ml of plasma in the first, second and third trimester, respectively [26]. Syncytin-1 TM subunit (which carries the ISD) in placental exosomes is between 4–8% of total exosomal proteins, which means that during the second and third trimesters, the concentration of the bioactive Syncytin-1 TM in maternal plasma is around 783nM and 1.2μM, respectively. Therefore our use of Syncytin-1 recombinant ectodomain at 1μM is within the expected concentration found in circulating placental exosomes in maternal blood, with the potential to interact with the maternal immune system. We sought to establish whether human syncytin-1 could impair maternal immune responses. Firstly we identified that exosomes isolated from placental explant tissue and maternal blood contained syncytin-1. We then determined that isolated syncytin-1 modified maternal immune responses, through inhibiting the release of tumour necrosis factor-α, interferon (IFN)-γ and CXCL-10 from whole blood following in-vitro stimulation with lipopolysaccharide[9]. The presence of syncytin-1 in placental exosomes provides a means of reaching and interacting with maternal immune cells and represents a novel mechanism of endogenous retroviral mediated immunosuppression that may be relevant for immune tolerance and the undesirable increased susceptibility to infection.

Dendritic cells are key regulators of immune responses, and can promote or suppress T-cell responses depending on the circumstances[5]. This feature of DCs relates to their ability to integrate a diverse array of incoming signals, and then to direct an appropriate adaptive T-cell immune response. The important role of DCs in promoting maternal tolerance in pregnancy has only recently been examined. As pregnancy progresses through the third trimester, these cells undergo changes that promote a state of reduced responsiveness. Monocytes from pregnant women were found to have differentiated into less phenotypically mature DCs and express lower levels of CD80, CD86 and HLA-DR molecules compared with non-pregnant women[27]. In response to inflammatory stimuli monocyte-derived DCs from pregnant women, up-regulated CD86 more than CD80, and secreted less IL-12p70 but more IL-10, compared to cells from non-pregnant controls[27]. This implies the DCs from pregnant women have a reduced ability to stimulate a TH-1 immune response, skewing it towards a TH-2 like response that is less capable of protecting against viral infection. Furthermore factors released from the trophoblast *in-vitro* have also been shown to down regulate innate immune responses from mouse DCs and natural killer (NK) cells[6]. Consistent with this we have also shown an impairment of the innate immune response to influenza in pregnant women[21]. pDC (CD303(+)/CD1c(-) PBMCs) percentages were lower in pregnant compared with non-pregnant women (P <. 05), while following H1N1/09 infection, pDCs from pregnant women showed higher expression of CD86 (P <. 01), HLA-DR (P <. 001), and the negative regulator PDL1 (P <. 001) compared with non-pregnant women. These PBMCs also demonstrated reduced release of IFN-α, IFN-γ and IL-2 in response to H1N1pdm09. There was also reduced cell mediated immunity with increased PD-1 expression on CD8 T cells[21]. Our current findings now demonstrate that exposure of PBMCs to syncytin-1 specifically impairs the early innate immune response to H1N1pdm09 with reduced release of IFN- α and IFN-λ from pDCs. Mouse models of influenza clearly demonstrate that pDCs are recruited to the lungs early in influenza infection, are an important source of early IFN-α and also regulate an early robust anti-viral T cell response[28]. Using a pDC deficient mouse, while the immune response to virus was delayed, an effective T cell specific response still occurred allowing effective viral clearance[28]. Therefore in the case of pregnancy, impaired pDC responses may not be enough alone to explain the susceptibility seen in pregnant women to influenza.

In the case of influenza infection, animal models show that clearance of infection requires a robust Th1 immune response against the virus[29]. The optimal Th1 response consists of virus-specific IFN-γ-secreting CD4 T cells and cytotoxic CD8 T cells that lyse virus-infected cells[30]. We have assessed the early immune response of PBMCs to influenza in our model and the response consequently at the 48 hour time point has predominately been from pDCs. A small number of CD4+ T cells were responsible for the release of IFN-γ in response to influenza and there was a trend to reduced release with syncytin-1. Optimal activation of CD4 cells are likely to require a longer incubation period with influenza and syncytin-1.

The pregnancy-associated susceptibility to intracellular pathogens has been classically attributed to a shift in helper T cell differentiation from a Th1 to the Th2-dominated response required for maintaining pregnancy[31]. Recent work has now shown that the requirement for expanded immune tolerance during pregnancy is more specifically linked with the expansion of maternal TReg cells. For example, circulating maternal Foxp3+ TRegs expand and at peak mid-gestation levels are 50 percent higher compared to the non-pregnant state[32]. More recently this TReg activation and subsequent immunotolerant phenotype has been shown to be regulated by the placenta[33]. Maternal TReg expansion required for sustaining pregnancy then may create naturally occurring deficiencies in host defence. The expansion of immune-suppressive Foxp3+ regulatory T cells (TRegs), which occurs physiologically during pregnancy or when experimentally induced in mice, has previously been shown to cause enhanced susceptibility to *Listeria* and *Salmonella [34]*. Susceptibility was reduced with TReg-ablation. Importantly, the sustained expansion of maternal TRegs was essential for maintaining immune tolerance to the developing fetus because even partial transient ablation of Foxp3-expressing cells fractured maternal tolerance to fetal antigen and triggered fetal resorption. We now show that exposure of PBMCs to syncytin-1 led to a significant release of IL-10. This release was predominantly from pDCs and to a lesser extent TReg cells. Increased IL-10 plays an important role in activating and maintaining TRegs[35] especially in local tissues, with TReg activation and differentiation occurring via Notch dependent signaling and IL-10 exerting a positive autocrine effect on TRegs[36]. In addition IL-10 has well described immunosuppressive effects on CD4+ cells, inducing anergy and a reduced ability to release cytokines[37]. Our data show that syncytin-1 has the ability to impair the early innate antiviral responses as well as the induction of a robust Th-1 response that is required to efficiently clear infection. However while our results may be sufficient to explain enhanced susceptibility to influenza in pregnancy, syncytin-1 appears to also play a more sinister role in acutely worsening the acute inflammatory response.

Influenza, including infection with H1N1pdm09, usually does not cause severe infection, with an overall case fatality rate of 0.5%. However, a small number of individuals are more severely affected, requiring hospitalisation, and of those 9–30% will be admitted to the intensive care unit (ICU), usually with severe pneumonia and systemic inflammation[38]. This response has been seen in other causes of acute severe viral pneumonia such as SARS[39] and with the highly pathogenic avian H5N1 influenza[40]. In these severe cases there is evidence of a dysregulated immune response characterised by severe lung immunopathology and the intense release of acute inflammatory cytokines, suggestive of an inability to switch from an initial innate immune response to an effective adaptive immune response. Certain influenza viruses specifically induce this response, with mouse models of H5N1 and the 1918 pandemic H1N1 influenza strain both showing intense neutrophilic lung infiltrates[41], the consequences of which may contain the virus, but with the potential to produce severe acute lung injury. In those with severe HN1N1pdm09 there is similar evidence of an exaggerated acute response. Lee et al[42] compared 63 hospitalised severe H1N1pdm09 patients to 53 individuals with seasonal influenza and found evidence of an hyper-activated pro-inflammatory response characterised by elevated IL-6, and a suppressed adaptive Th1/Th17 response, with reduced CXCL-10, IFNγ and elevated IL-10. In addition elevated IL-6 was an independent predictor of admission to ICU and correlated closely with ex-vivo cytokine responses from PBMCs. Pregnant mice infected with H1N1pdm09 developed acutely increased IL-6, MCP-1 and KC (the mouse equivalent of CXCL-8), which was associated with an intense neutrophilic infiltration of the lung that lead to more widespread epithelial damage and a failure to repair damaged tissue. In addition the adaptive immune response was impaired with an increase in alternatively activated macrophages, and while no difference was seen CD8 T cell responses, the pregnant mice had higher numbers of TReg cells present in the bronchial alveolar lavage fluid[43]. It has recently been shown that a similar picture occurs in human pregnancy associated with H1N1pdm09 [44] where it has been found that CD69 on T lymphocytes and the TNF-α, IL-1β, IL-6 and IL-10 sera cytokines as well as CXCL8 were increased in H1N1pdm2009 virus-infected women. Also, pregnant women have increased NK- and T-cell cytokine and chemokine production in response to influenza virus in vitro, particularly H1N1pdm2009 virus, without a significant change in NK-cell, CD8+ T-cell, or CD4+ T-cell frequencies [45].

Our results suggest that the response in pregnant women to HN1N1pdm09 may be due to syncytin-1. Treatment of PBMCs with syncytin-1 alone was sufficient to increase the release of IL-1β and IL-6, with further small increases seen following exposure to H1N1pdm09. The release of IL-1β implies that treatment of these monocytes with syncytin-1 was sufficient to trigger the inflammasome activation. Monocytes synthesise and release IL-1β as inactive cytoplasmic precursor into the cytoplasm, and it is processed into biologically active forms in response to various pro-inflammatory stimuli (including viruses) by the cysteine protease caspase-1[46]. While influenza infection can directly activate monocytes, macrophages appear to require priming with another pro-inflammatory signal before an optimal response is seen following infection[47]. Syncytin-1 could be that signal. In pregnancy the presence of syncytin-1 may not only impair antiviral interferon responses from monocytes and DCs, but also lead to heightened activation of the inflammasome, with an exaggerated systemic inflammatory response, replicating that seen in those with severe H1N1pdm09 infection. The negative impact that such a response could have is considerable, as recent evidence suggests that genetic variants of IL-1α and IL-1β may exert a substantial impact on the susceptibility of H1N1pdm09 infection[48].

Retroviral infections often induce immunosuppression in both humans and animals and have been related to a highly conserved sequence within the retroviral envelope, the transmembrane subunit protein, called the immunosuppressive domain (ISD)[49,50]. Experiments using a 17-amino acid synthetic peptide dimer (CKS-17) with the consensus ISD sequence have induced immunosuppression both *in vivo* and *in vitro* on lymphocytes, monocytes, macrophages, natural killer and natural killer T cells, including the modulation of Th1 and Th2 immune responses. CKS-17 has been shown to inhibit the Th1 cytokines TNF-α, IFN-γ and IL-2 along with an upregulation of IL-10[49,51–53]. It has also been shown that the recombinant transmembarne protein gp41 of the human immunodeficiency virus HIV-1 and a synthetic peptide with its ISD sequence, up-regulate the expression of IL-6, IL-10 and IL-1β in a dose-responsive and sequence specific manner in human PBMCs[54,55]. Our results are consistent with this. When PBMCs from non-pregnant women were exposed to syncytin-1, an endogenous retroviral envelope protein, the resulting response was entirely consistent with that described for retrovirus mediated immunosuppression.

This study demonstrates that PBMCs from pregnant women exhibit an impaired early antiviral response to influenza. We now show for the first time that this response can be induced in the PBMCs from healthy women by exposure to the placental derived protein syncytin-1. Syncytin-1 exposure leads to a marked reduction in pDC release of IFN-α/λ to influenza, but it also stimulates enhanced release of IL-10 with the potential to further impair adaptive responses as well as enhanced release of IL-6 and IL-1β both of which potentially will worsen systemic inflammation. This is the first study to identify a direct link between a placental derived protein and suppressed maternal immunity. Given the risk posed by both seasonal and pandemic influenza, as well as other intracellular pathogens, to pregnant women this is a very important step forward and potentially identifies a target that could be manipulated to improve maternal and fetal outcomes globally during future pandemics.

## Supporting Information

S1 Fig Dose response of syncytin-1 in non-pregnant women. The response of PBMCs from non-pregnant women to different doses of syncytin-1 was assessed after 48 hours of exposure. PBMCs from non-pregnant vaccinated (NPV) women were exposed to increasing doses of syncytin-1 (1nM, 10nM, 100nm, 1μM and 5μM). Levels of IFN-α (a), IFN-λ (b) and IL-10 (c) in cell culture supernatants were measured by cytometric bead array. ANOVA (Holm-Sidak) **** *P* < 0.0001, *** *P* < 0.001, * *P* < 0.05.
(TIF)

S2 Fig Flow cytometric analysis of PBMCs showing that CD4 cells are responsible for the release of IFN-γ and to a lesser degree, IL-10. PBMCs were infected with H1N1pdm09 MOI 0.1 for 48hrs with and without syncytin-1. The cells were then subjected to surface and intracellular cytokine staining and flow cytometry to determine cell populations and cytokine release. The CD4 population was determined using CD3+/CD45+ cells which were also CD4+ and CD8-. This population was analysed for cytokine release using intracellular staining and the % of positive cells for IFN-γ (a) and IL-10 (b) was calculated. NPNV = Non-pregnant and non-vaccinated n = 2.
(TIF)

S3 Fig Dose Response of H1N1 in non-pregnant PBMCs. The response of PBMCs from a non-pregnant woman to different MOIs of H1N1pdm09 was assessed after 48 hours of exposure. PBMCs from a non-pregnant vaccinated (NPV) woman were infected with H1N1 of increasing doses (MOI 0.1, 0.5, 1 and 2) for 48hrs. Levels of IFN-α (a) and IFN-γ (b) in cell culture supernatants were measured by cytometric bead array. Cell viability was measured by Annexin V-PE and flow cytometry and percentage of necrotic, apoptotic and viable cells were separated (c).
(TIF)
