# Supplementary figures and images for: The Placental Protein Syncytin-1 Impairs Antiviral Responses and Exaggerates Inflammatory Responses to Influenza
Source: PLoS One. 2015 Apr 1;10(4):e0118629. doi: 10.1371/journal.pone.0118629 (PMC4382184; doi:10.1371/journal.pone.0118629)

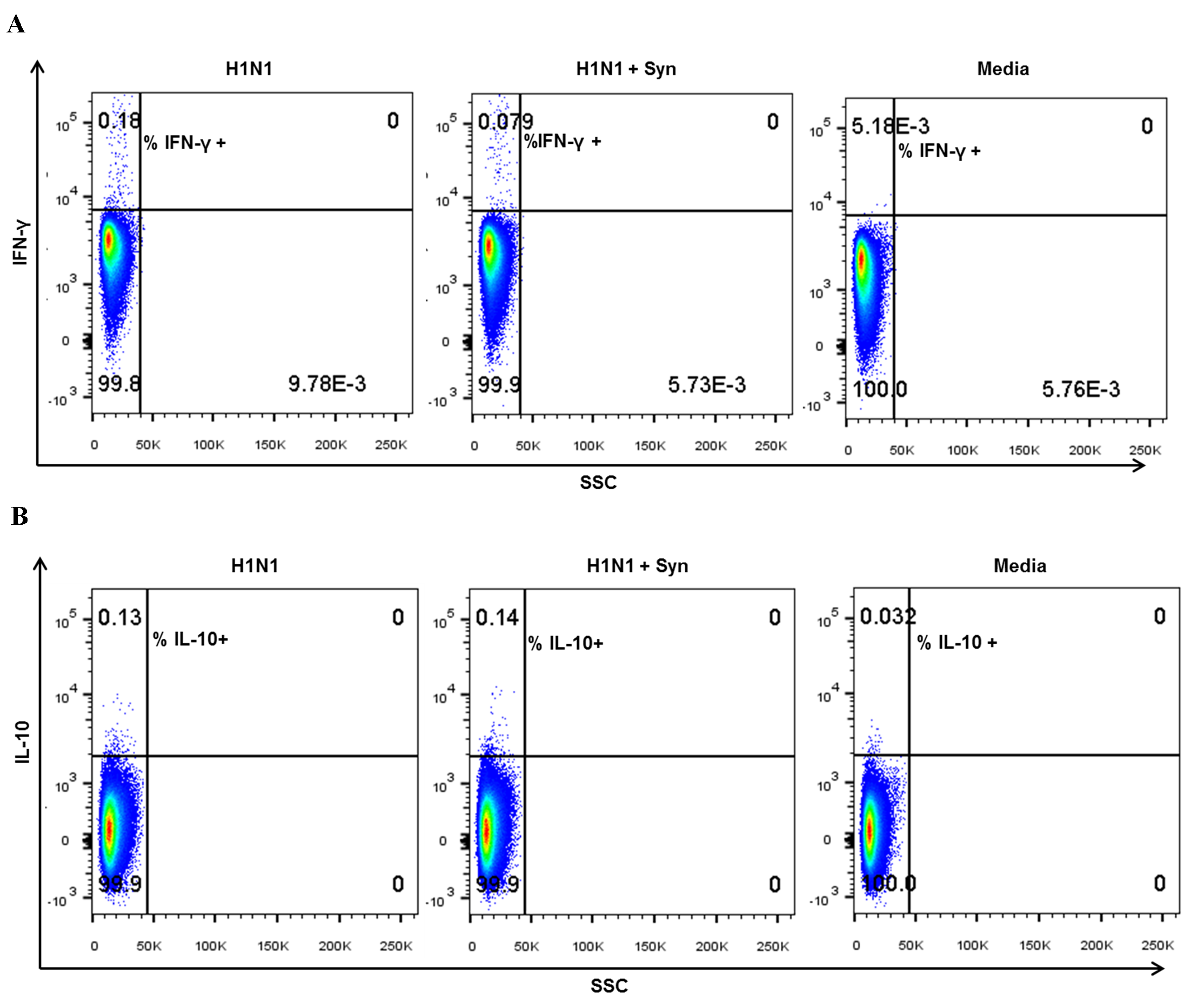

Supplement: S2 Fig — PBMCs were infected with H1N1pdm09 MOI 0.1 for 48hrs with and without syncytin-1. The cells were then subjected to surface and intracellular cytokine staining and flow cytometry to determine cell populations and cytokine release. The CD4 population was determined using CD3+/CD45+ cells which were also CD4+ and CD8-. This population was analysed for cytokine release using intracellular staining and the % of positive cells for IFN-γ (a) and IL-10 (b) was calculated. NPNV = Non-pregnant and non-vaccinated n = 2. (TIF) [file pone.0118629.s002.tif]
